# Computational design logics for bio-based design

**DOI:** 10.1007/s44223-022-00015-8

**Published:** 2022-09-26

**Authors:** Mette Ramsgaard Thomsen

**Affiliations:** CITA Centre for IT and Architecture, Royal Danish Academy, Architecture, Design and Conservation, Copenhagen, Denmark

**Keywords:** Computational design, Architectural intelligence, Functional grading, Integrated sensing, Adaptive fabrication, Bio design

## Abstract

This paper examines how the central contributions of the computational design field can be understood as central steppingstones into an age of sustainability to engage with new renewable, regenerative and restorative material systems. By taking departure in the conceptualisation of an extended digital chain by which architecture can address fabrication at the low scales of the material, this paper asks how these methodological innovations can be transferred to new questions arising from a bio-based material paradigm. The paper outlines the three central contributions of the computational design field: advanced information modelling, functional grading and integrated sensing, and suggests how these can be extended to allow new means of instrumentation for bio-based materials characterised by the heterogeneous, the behaving and the living.

## Introduction

**Fig. 1:**
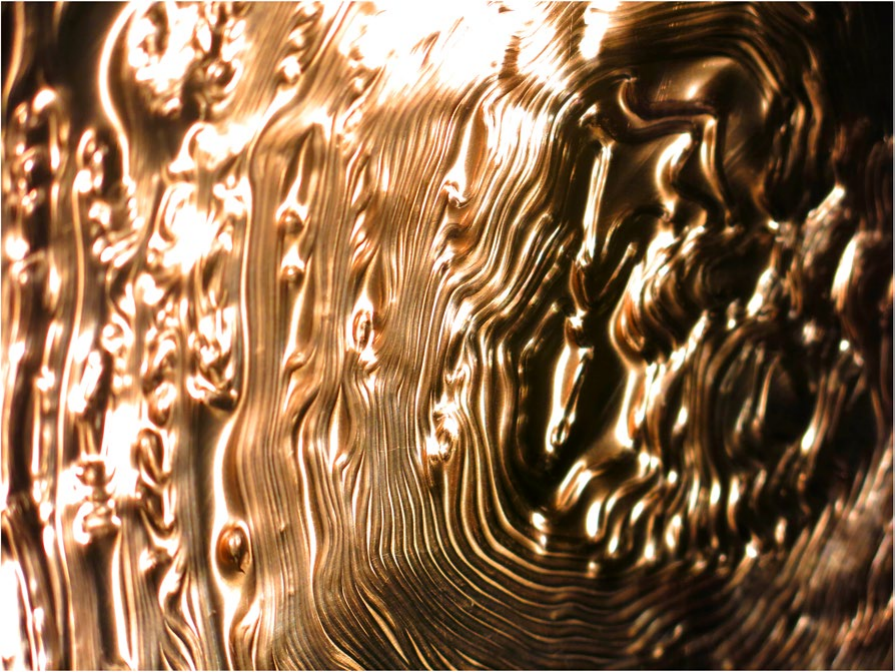
Copper Clad, Sydney, Australia. Incremental sheet forming processes changing the microstructure of the material. CITA 2018

Architectural intelligence frames a broad territory connecting the computational, the automated, the augmented and the manual. With focus on new modes of production, it asks how the technologies of design, analysis, specification and fabrication can be connected and extended allowing stronger correlations between the practices of design and making. As such, the term architectural intelligence is directed towards the core of architectural thinking. If architecture as a tradition is a prescriptive practice associated with acts of building representations, it is always fundamentally focussed on the ways in which these representations correlate to material practice through material designation, specification and detailing. Even in its most speculative renditions, architecture is always about material thinking (Fig. 1).

The arrival of computational design systems has challenged the way this correlation is understood. Rather than relying on acts of interpretation, the direct interfaces between design and fabrication allow the model to become an instruction directly steering processes of materialisation. During the last 15 years new *extended digital chains* (Thomsen & Tamke, 2020) have developed ways of informing design intent with contextual analysis through data rich practices, interfaced our models with live sensor based information flows, incorporated analysis and simulation enabling the rethinking of structural systems and opened up to new creative horizons examining how material systems can be hyper specified and functionally graded in order to engage with and steer material performance at multiple and concurrent scales. The underlying ethos of this increasingly consolidated field is one of optimisation. The theoretical position being that building intelligence is allied with material reduction and that computational design systems allow us to rethink the infrastructures of the design model in order to *build smarter with less*. However, as our contexts change and we venture deeper into the environmental crisis with its repercussions on ecology, resource and social equity, it is clear that we need to readdress the uniformity of this position and ask how emerging regimes of renewable, regenerative and restorative material systems that are energy efficient, CO2 reduced and pollutant aware can challenge and change computational design.

This position paper presents an overview of the field with the aim to situate, discuss and give perspective on the current challenges emerging within the field of computational design. With special focus on the changing circumstances instigated by sustainability and climate change, it identifies the field’s key methodological contributions: advanced information modelling, functional grading and adaptive fabrication and asks how these can be reimagined in a new sustainable age. By pairing these contributions to a new era focussing on bio-based resources, the paper asks how the particular properties of bio-based materials present a series of disruptions to this methodological framework. It presents how these challenge the fundamental value proposition of industrialised building systems by positioning a new value system that accentuate the heterogeneous and the behaving, instead of predicating design on an idealisation of permanence and stability. The paper positions computation and information rich design as key methodologies for emerging practice, bringing forth a vision of an extended digital chain in which presumed linearity is dismantled in favour of cyclical methods defined by feedback between concurrent processes of specification and fabrication and nonlinear temporalised modelling practices driven by situated sense data.

**Fig. 2:**
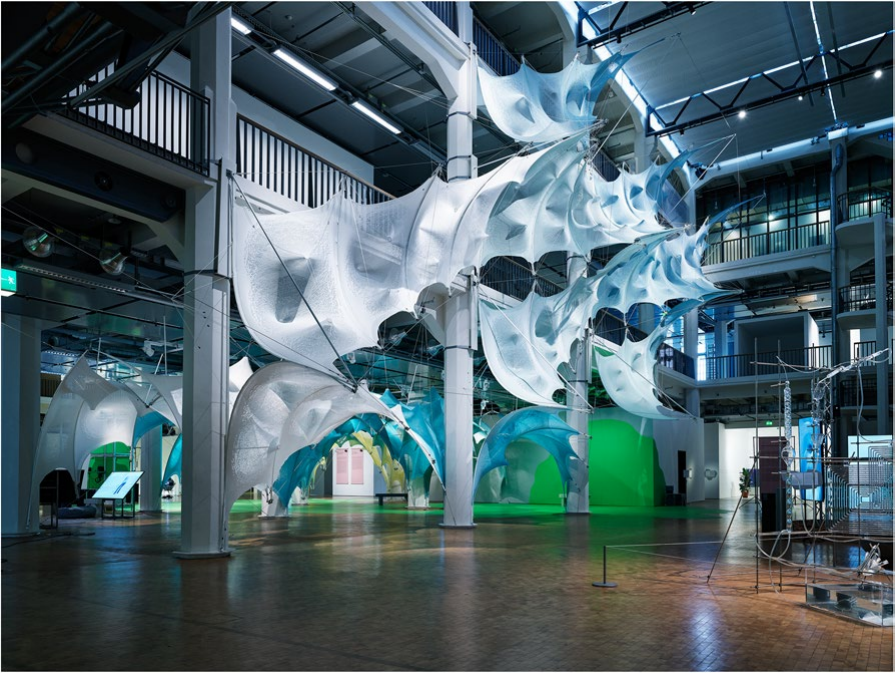
Zoirotia at ZKM, Germany. Zoirotia examines the making of graded membranes using knit as a substrate. CITA 2022

The paper argues the evolving dimensionality of this argument through a breadth of projects from CITA’s (Centre for IT and Architecture) research practice through which particular concepts and drivers have been identified. These projects are developed employing an inductive ‘research-by-design’ method focussed on design-led physical experimentation and full-scale prototyping. The method foregrounds the creation of prototypical workflows (Poinet, 2020) that probe the computational infrastructure and fabrication methods enabling novel material practices. Research-by-design positioning allows the research inquiries to simulate the network of interconnected practice, cross technological solutions and inter-scale material thinking that make up design to fabrication workflows in the built environment. The material experiments generate shared empirical data that can be tested, analysed and evaluated by the interdisciplinary research teams that are engaged in the research creation. These inductive processes are met with deductive research processes of theory building. In these processes key concepts defined through the projects are connected across project activities constructing the overarching theoretical positioning (Fig. 2).

## CITA: Centre for IT and Architecture

CITA (Centre for IT and Architecture) is a research centre at the Royal Danish Academy, Architecture, Design and Conservation. Founded in 2005, CITA examines how new information and communication technologies fundamentally challenges architectural practice as a tool for design, fabrication and communication. CITA’s practice has been central in forming the field of computational design from its early outset in digital crafting and fabrication to its current focus on intelligent, automated and augmented design systems. CITAs work has a strong cross-disciplinary focus consolidating collaborations with interdisciplinary partners from the fields of engineering, design, robotics, material science and biology. The work is intentionally inter-sector based. By merging ground research goal with applied research agendas, we advocate knowledge creation across different sites of production bringing together knowledge in academy and industry.

Research in CITA is undertaken through a broad portfolio of project from large scale framing projects such as Eco-Metabolistic Architecture (Thomsen & Tamke, 2022), Fungar (Adamatzky et al., 2019), Complex Modelling (Thomsen et al., 2020) and Flora Robotica (Hamann et al., 2015), network and young research facilitation projects such as Innochain (Sheil et al., 2020) as well as small scale probes that enable novel ground research concepts and build new interdisciplinary collaborations such as BioLum (Thomsen et al., 2021). The strategic breadth of these activities allows for an active cross pollination between ideas, design strategies, modelling methodologies and fabrication.

The central remit in CITA over the last 17 years has been focussed on examining the potential of novel material systems and how the intersection between advanced computational design, sending, steering and robotic fabrication can unleash new creative material strategies. At the same time, these technologized investigations are always brought back to the architecture enquiry in which they are part. While researching and developing new methodologies for digital design and their associated technologies, our work also examines their expressive potential and underlying aesthetics, asking *what difference* technology makes. In this way, we strive to bring back potentially positivist technology-driven enquiries to a deep-seated ground-research enquiry that asks how the changing role of architecture representation fundamentally changes what architecture can be.

### Key contributions of computational design

Computational design culture is fundamentally aligned with industrialisation. If it, from its outset, has challenged standardisation and theorised the non-standard (Migayrou, 2014), its drivers lie with linking design agency to the processes of automation, mass fabrication and standardisation that have defined modern construction. Early proponents of the field highlighted the ability to link algorithmically driven design generation to the processes of mass customisation allowing for a richer, more expressive or more situated design language (Kolarevic, 2004; Carpo, 2011). The central contribution of the digitisation of architectural design has been the consolidation of a shared digital platform – a *digital chain* – interfacing information flows in order to optimise the processes of design, analysis, detailing, specification and fabrication (Thomsen & Tamke, 2020). In industry this has been driven on the one hand by the development of industry standards for linear modelling such as Building Information Modelling (BIM) and the IFC standards for interoperability that have enabled a systemic innovation of Architectural, Engineering, Construction and Operation sectors transforming how information is shared (Casini, 2022; Bolpagni, 2022)). These methods have facilitated an innovation of existing practice within industrialised building systems creating more efficient means of integrating environmental analysis, green energy analysis, crash detection analysis, quantity & cost analysis, operation and maintenance information (Meins-Becker et al., 2019; Chen et al., 2016; Yuan et al., 2018; McArthur, 2015) (Building Information Modelling, 2020). BIM designates a *linear and unified approach,* relying on the inscribing of building systems into information objects and the development a shared taxonomy. This effort has been paralleled by a *networked and circular approach* (Thomsen, 2016) in which digital design tools are more loosely and opportunistically interfaced with a host of programmes from neighbouring design and analysis fields as well as an innovative cross-over of industry- and grassroots driven breadth of plugins, apps and peripherals that bring algorithmic intelligence to the design space. In industry, both practices are firmly tied to industrialised building systems. Information modelling and computational design fundamentally corroborates the fixity of the industrial building systems and the material systems they employ asserting their inscribed ideals of standardisation, repeatability and an ideal of permanence.

In research, computational design and the digital chain has led to a rethinking of these axioms. The key driver has been the aim to optimise resource allocation. Fuelled by a general sustainability agenda, computational design has created the methodological framework to optimise the way we understand and use material. By addressing the general scientific problems of linking advanced material simulation to the design model and simultaneously addressing fabrication at the level of material composition, computational design research is rupturing our understanding of how material systems can be designed, fabricated and employed. Integrating advanced material simulation has led to the forming of a new set of digital-material relations that fundamentally conceive materials as *performative*; responding to stresses and changing behaviour in time. Fuelled by the creation of advanced computational design methods that enable *feedback* between otherwise discrete design phases of design, simulation and digital fabrication, and informed by the fields of aerospace engineering, robotics, computer science and technical textiles (Deleuran et al., 2016; Jipa et al., 2016; Thomsen et al., 2019), the emergence of concepts of material driven form-finding or morpho-genesis has brought about a new performative perception of materials (Loh et al., 2016; Menges, 2012; Oxman, 2012; Tamke et al., 2012). This has opened new research horizons aiming to steer material performance interfacing the low scale composition of materials with high scale performances of the overall structural system through advanced fabrication systems either through established CNC systems or through bespoke robotic tools reinventing material address. Large scale research efforts into different material practices such as concrete (Lloret et al., 2015; Méndez Echenagucia et al., 2019), masonry (Buchli et al., 2018), timber (Aldinger et al., 2020; Robeller & Weinand, 2016), steel panelling (Nicholas, Stasiuk, et al., 2016), textile membranes (Sabin, 2013; Thomsen et al., 2016) and fibrous shell structures (Dörstelmann et al., 2015) investigate advanced interfaces between design and fabrication effectively expanding the digital chain into material fabrication but also changing the nature of the agency and remit of the architect connecting the design of buildings and structures with the design of materials.

In this emerging scientific framework, materials are understood as structural compositions that pertain to inherent sets of properties and dynamic behaviours. The theoretical underpinning of the computational design field is that *by formalising these properties and behaviours and devising means of representing their inherent complexity, we can access their underlying compositional logics and thereby gain agency to their steering* (Thomsen et al., 2020). The extended digital chain is a central proponent of this theorem tying a performative design ethos to the ability to variegate material steering and enable functional grading for architectural materials. This new paradigm effectively challengesthe mass-fabrication logic of the standardised industrial series and enabled a rethinking and profound innovation of material systems.

**Fig. 3:**
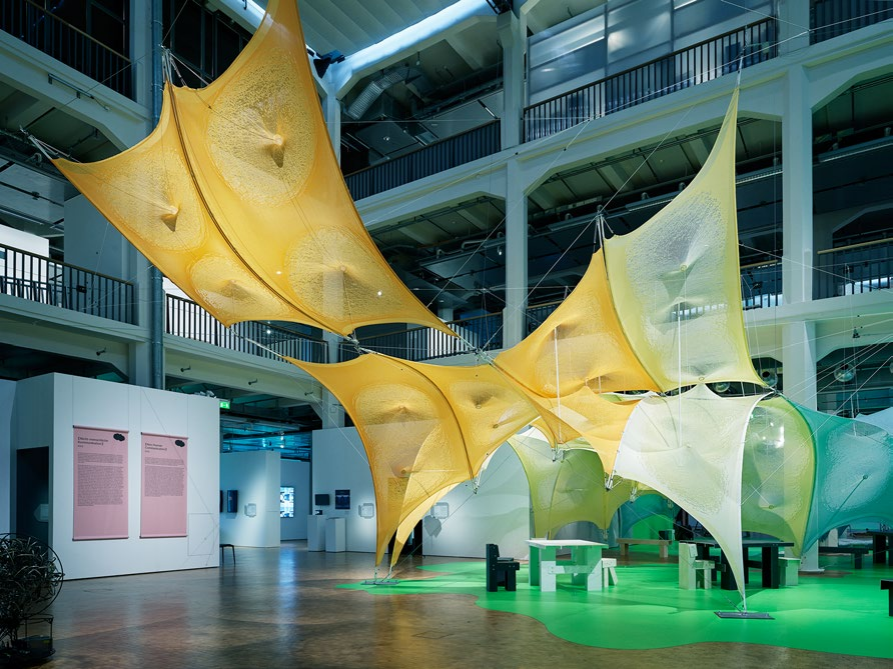
Zoirotia at ZKM, Germany. Colour is controlled by grading the duotone system of Jaqcard knit. The grading of intensity follows the structural performance as material extensions around the pre-tension points grade translucency. CITA 2022

The framework is progressed by the creation of three central methodological developments; networked modelling, functional grading and adaptive fabrication. These three key computational infrastructures are seen as a progression from the three underpinning axioms of linear design modelling, standardisation and mass fabrication that are the industry standard. The following detailed description outlines the core contributions of these methodological developments. They are seen as part of an *inclusive* framework (Fig. 3) which parallels and interacts with the continuation of industry standards. The paper argues for their contribution and set them up as key steppingstones for a breakthrough devising the necessary computational infrastructures for a bio-based material paradigm.

#### Networked modelling – characterising, simulating and predicting material response for architectural construction

If architectural representation is traditionally understood as a prescriptive practice following a continual descent through the scales in which increasingly detailed information moves from planning, designing, specifying and detailing, then computation fundamentally challenges this implied linearity. The fundamental contribution of computational design is *feedback*. Feedback is present in multiple forms: in the design chain enabling better decision making, between partners and most innovatively to inform design decision making through understanding material performance. Foundational to the practice of form-finding (Lewis, 2003; Veenendaal & Block, 2012), the ability to characterise, simulate and predict material performance allows designers to actively inform design with an in depth understanding of how stress-strain relationships impact on structural behaviour and how this in turn influences form. In research, this correlation between design and material behaviour has founded new research directions that seek to optimise material deployment and rethink structural design through the innovation of new hybrid systems (Gengnagel et al., 2013; Quinn et al., 2016). Where, early formative projects piloted means of integrating the structural simulation of standardised building materials with computational design environments (Fleischmann et al., 2012; Tamke et al., 2012), advances in the field have extended these methods with increasingly low scale material simulations permitting the analysis of non-standard and functionally graded material systems. The insight in the field is that different scales of material address are connected, that they are concurrent as opposed to sequential, and that agencies across the scales affect each other in manifold ways.

In CITA, our contribution to the field has been the creation of a multiscale modelling framework interfacing a breadth of differentiated models that span between the descriptive and design intent developing, the mechanistic and simulation bound that enables performance characterisation at multiple and concurrent scales of intervention and the predictive-statistical based on data from either internal-generative processes or external-sensor based processes. The modelling framework extends the potential of the information model while disrupting current building practice’s strategy of separating processes into clearly defined hierarchies with deliberately phased clean cut off points. In projects such as Complex Modelling, we have examined how an expanded understanding of the information model as a network of highly specialised models, that pass information to each other can allow design to happen in parallel with and as a function of understanding material performance. Here, a central insight is that information needs to flow in multiple directions and that ‘handshakes’ need to be strategized to allow information interfaced across the digital chain (Nicholas, Zwierzycki, et al., 2016). Networks of models can be arranged sequentially or in parallel with feedback travelling up or down stream through the scales of design agency. Analysis and simulation can take place at multiple concurrent scales passing information to each other in order to parameterise decision making. They can be in part automated, triggering processes in which new models are generated in order to create optimisation regimes. As such, models are multifarious; they occur in parallel and across a discretized time base (Thomsen et al., 2020).

A key consequence of this method is the expansion of the model’s representational logic changing the model’s central axiom from one of ‘how things look’ to ‘how things behave’. Integrating simulation into design models and interfacing them in ways that allow design to take full advantage of a material systems internal behaviours, challenges the boundaries of the architectural model and drives it towards a mechanistic modelling logic. In difference to passive descriptions of active systems, mechanistic model is active: it can be built, run, followed step by step and calibrated to achieve a higher level of accuracy (Glennan, 2005). In this way, mechanistic models describe a system of parts and the underlying mechanisms that govern the interaction between parts and its containing environment and thereby define the behaviour of a system. The focus on behaviour deeply challenges the traditional linearity of design process. Rather than emphasising scale it places *graded fidelity* as a key property of the information model (Thomsen et al., 2020). If behavioural analysis takes place at multiple concurrent scales of the model, it is with a strategically varied degree of fidelity, it’s coarseness or fineness depending on the processes in which the information is instrumentalised.

This fundamental ethos of advanced information modelling makes it anything but unified. Instead, the different concerns of each part model are celebrated enabling highly specialised and domain specific information to enter design decision making. In CITA, and through our collaborations, we have further expanded this thinking by opening up the boundaries of the mechanistic model to include statistical-predictive methods. Mechanistic and predictive models are often understood as contrary modelling practices. Mechanistic models are based on data that can be referenced, validated and verified independently to describe phenomena, then statistical-predictive models aim to correlate data points that best describe the data itself (Baker et al., 2018). New approaches find ways of hybridising them. In CITA we expand our multiscale modelling method to include prediction based on both internal generated design data and external data from environment or steering in order to enrich the modelling landscape and inform design decisions.

**Fig. 4:**
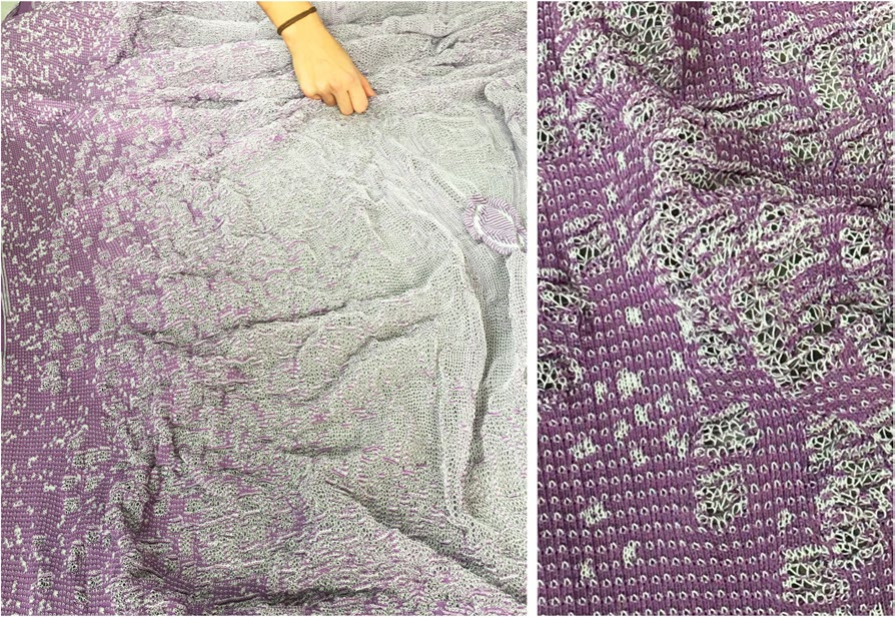
Grading strategy for knitted membranes and their simulation. Knitted membrane can be simulated as a cablenet-like quad-based mesh, as it represents the UV-directionality of the knit and the pliability of the surface under the loads. For representing graded properties, each mesh segments is assigned a variagated stiffness value, which results in differentiated contraction (or expansion) of the digital mesh and represents graded property of the membrane surface. CITA 2022

What is arrived at is a new practice of advance information modelling in which architects can inform design from a breadth of concurrent and at times competing behavioural analyses taking place at multiple scales and in discrete time. It provides methods for a new design practice that foreground design as a *steering of interactions* between sub parts that together are the *design system* (Fig. 4).

#### Functional grading for architecture and construction

**Fig. 5:**
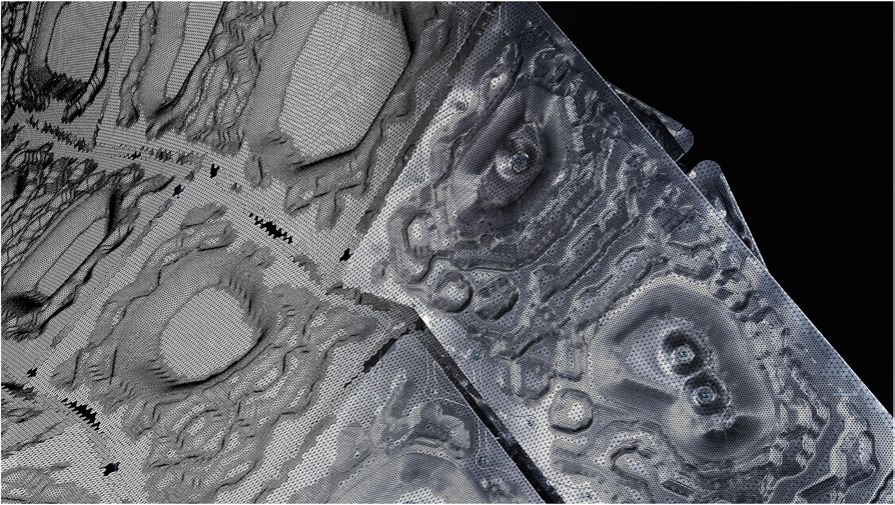
A Bridge Too Far, Copenhagen. The incremental sheet forming of the panels is learnt and fed back to the control system allowing for adaptation for the dynamic behaviours of spring back. CITA 2017

The second methodological development undertaken by the field of computational design has been the transfer and repositioning of conceptions of functional grading for architecture and construction. New computational design practices foreground designed material heterogeneity as an opportunity for optimisation. Functional grading in architecture is tied to a behavioural understanding of materials and a central component of the idea of material steering. If advanced information modelling allows a higher degree of sensitivity to the interacting subparts of the design system, then functional grading is the instrumentation of its response. It is through practices of hyper-specification of materials and their grading in response to varying performance criteria imposed by context or function that architects gain agency within the extended digital chain (Fig. 5).

Functional grading is a highly interdisciplinary set of methods for varying the structure and composition gradually over volume (Naebe & Shirvanimoghaddam, 2016). Arriving from the fields of aircraft and aerospace industry as well as material science, functional grading in architecture is driven by the opportunity to interface and control material deposition. Efforts for functional grading have taken place across a range of material systems including concrete (Herrmann & Sobek, 2017), fibreglass (Nicholas & Tamke, 2013), fibre winding (Estrada et al., n.d.) and various strategies for 3D printing (Doubrovski et al., 2015; Royo et al., 2015). Here, material fabrication systems are precision steered to strategically allocate material in order to fulfil highly localised performance criteria. The grading can take place by both guiding material placement and structure as well as changing their composition. Strategies can operate through the deliberate interchanging of materials with given properties or through the control of the constituent material parts and their continual change.

**Fig. 6:**
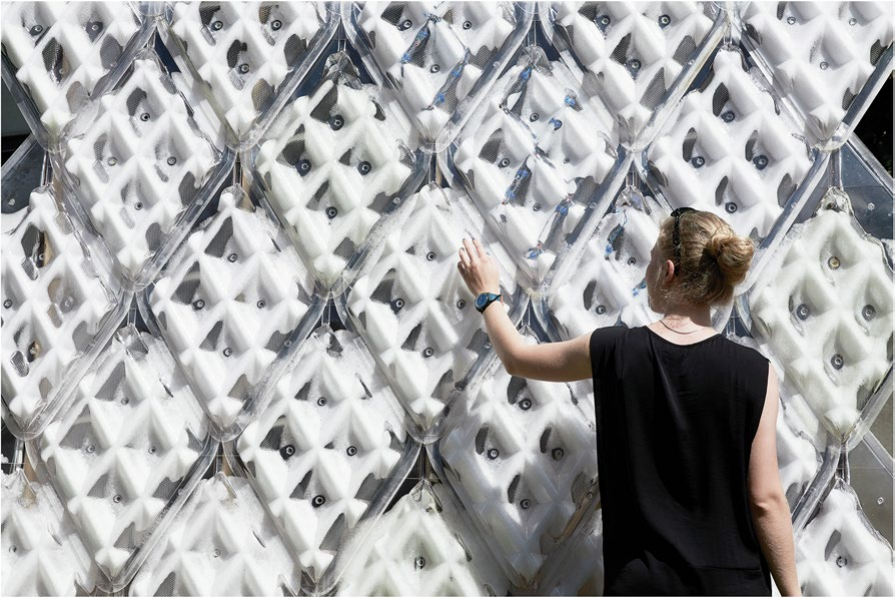
PCM Façade, Copenhagen. Panels are differentiated to allow different environmental exposure thereby controlling the melting points of the waxes. CITA and Billie Faircloth, Kieran Timberlake Research, 2017

In CITA, focus on material hyper-specification has matured methods by which to interface low scale material address to steer material behaviour. This work brings concepts of form-finding together with an idea of material steering in which a fundamentally heterogeneous materiality is interfaced with design intent. Early work examined the layup of graded fibreglass elements in active bent structures (Thomsen et al., 2015). By using standard fibreglass strips, the number of layups are differentiated resulting in the grading of material stiffness across the structure in turn allowing us to tune the active bending of the overall structure. These early examinations questioned how practices of form-finding and the theory of material behaviour gains new agency and a profound circularity in the design space when *materialising design becomes interfaced with designing materials* (Nicholas, 2013) (Fig. 6).

These concepts are matured in a series of projects looking at materially heterogeneous structures. By employing diverse material systems and, as such, fundamentally different systems of design, simulation and fabrication, these investigations develop methods for employing new degrees of material address the micro scale of materials to steer their behaviour and performance. Two central strategies can be exemplified in our work on incremental sheet forming (Nicholas, Stasiuk, et al., 2016) and knitted membranes (Thomsen et al., 2016). Both project strands use standardised material substrates (metal panels and high-performance yarns) to create highly graded material systems. In the incremental sheet forming projects, standard metal sheets are locally deformed affecting the material microstructures. The interaction across scales of performance is central. The forming process simultaneously hardens and thins the material creating competing and concurrent processes of strengthening and weakening the sheet material. At the same time, the material is three dimensionally forming creates a local shaping that increases the stiffness of the elements. Finally, the elements are brought together into a structural arch creating orchestrating an overall structural performance. Our investigations examine how to steer design agency across these differentiated material scales and how to enable feedback across them allowing us to adaptively parametrise performance across a highly graded material system.

**Fig. 7:**
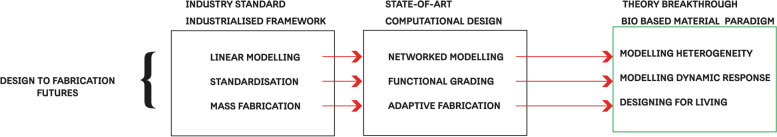
Diagram outlining progression of key computational infrastructures from industry standards, to state-of-the-art in the Computational Design field to a new bio-based material paradigm. The framework is understood as inclusive driving a plurality of design to fabrication futures that need to co-exist to enable a sustainable building practice

In the knitted membrane projects, we address the scale of the individual stitch to grade material performance. The graded knitted membranes are employed as tensioning elements in a series of bending active arches. Here, the material substrate, the yarn, remains continuous but the system of structures and their local differentiation allows the variegation of the structural locking point. Knit structures can change in simple stitch size or in the actual system of looping creating more or less deformable structures as well as steering its inherent anisotropy. In projects such as Isoropia and Zoirotia, it is not the stitch size alone but the structural interconnectivity that is graded. Here, we interchange between different stitch systems allowing a looser system of stitches in defined patches around the pre-tensioning points. This integrates a material three dimensionality in turn driving the depth of the pre-tensioning resulting in a deeper architecture of the bending active system and therefore better structural performance. In Zoirotia (2022), this method is further expanded by employing a system of controlled unravelling. Knitted in a double jacquard structure we strategically introduce drop stitches in particular zones that allow the structures to unravel under tension in defined areas. As the structure is installed, this unravelling process is self directed, loosening the membrane around the pre-tension points to a form-found equilibrium. The material process is further accentuated through the introduction of a duo tone colouring of the patches. The unravelling process changes the colour intensity of the structure and produces a graded localised translucency (Fig. 7).

**Fig. 8:**
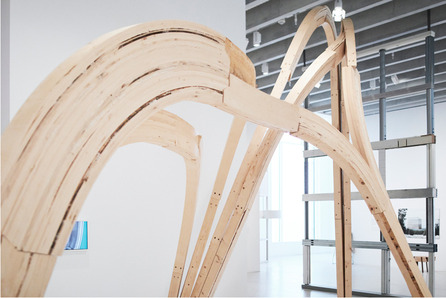
Rawlam, Bildmuseum, Umeå, Sweden. CITA, 2022

The creation of methods for functionally grading create step changes in the conceptualisation of architectural materiality that fundamentally challenge industrial fabrication paradigms. Exceeding existing dichotomies of the standard and the non-standard as well as the driving concepts of material optimisation through systems of material reduction, functional grading presents material as a plastic entity to be continually modified. What results is a highly performance driven heterogeneous material with differentiated behaviours that directly addresses the particular load cases or other design performances implicated. Albeit seated within industry driven methods of fabrication automation; the robotic steering of incremental sheet forming or the CNC knitting systems, functional grading expands the idea of the ‘series’ asking how materials themselves can be thought of as fundamentally individualised as a response to their use case (Fig. 8).

#### Integrating sensing for adaptive fabrication

The third contribution of the computational design field is the integration of sensing into the design to fabrication workflow. In creating the extended digital chain and innovating new classes of highly performance specific heterogeneous materials, the need to find novel ways of material characterisation arises. If the traditions of industrial fabrication reference standardised data sets that are validated and verified independently, then these new bespoke material practices that instrumentalise the active behaviours of materials necessitate new ways of understanding and predicting material performance in respect to its design implementation as well as the ways in which they behave under fabrication. Here, the integration of sensing into the digital chain interfacing design model, its processes of specification for material grading and the fabrication process itself become central infrastructures for the extended digital chain.

The integration of digital fabrication into the digital chain has meant the integration of a new level of material knowledge into the architectural representation (Thomsen, 2011). If the architectural representation traditionally is understood as fully prescriptive, relying on a set of notational norms that communicate material allocation, then the digital model as interface to fabrication incorporates instructions to the machine. In the outset this fabrication information can be simple; coordinates for CNC address or G code for milling or print paths. However, these methods assume a homogeneous and stable material address. As material systems become more complex, a new communication between the model and the fabrication system becomes necessary that is able to feedback an active characterisation of their behaviour; the material springback, bending or creep. The ability to use sensing to establish novel adaptive fabrication systems has informed research into architectural fabrication systems across multiple material practices. In fibre winding, sensing has been used to calibrate the correlation between the position and contact force allowing a robot to adapt its own code to place fibres on a flexible pneumatic membrane (Vasey et al., 2015). For timber, computer vision has been used to accommodate spring back and the irregularity of wood bending (Schwartz et al., 2014) as well as calibrate the robotic force of automated timber processing such as carving (Brugnaro & Hanna, 2017). Here, sensing is used to transfer from human action to machine action employing multiple sensors driving location (motion capture) and force (load sensing). In experiments with clay extrusion responsive systems are set up between the tracking of the extrusion system and its control allowing online adaptation (Bilotti et al., 2018).

In CITA, our contribution to the field has been twofold: in process and post process. As part of the sensing *in process* projects, we have developed methods of employing off- and online data to train fabrication and automate models to capture, steer and continually update fabrication data in response to local deviation. This is for instance exemplified in the A Bridge too Far project, in which incremental forming is understood as an accumulative process in which data is collected, analysed during forming and used to train a predictive model driving the fabrication control (Zwierzycki et al., 2018). In this methodological setup, fabrication process is understood as a correlation between the active behaviours of the material and the sense data drives an adaptive fabrication logic in which the model incrementally becomes increasingly intelligent and specific to the particularity of both material system and fabrication system.

A second strand of integrating sensing strategies has been to use material sensing post process. Here, sense data is gathered from prototypical elements across longer life spans to inform performance. An example of this is the PCM Façade project, a collaboration with Kieran Timberlake Research (Faircloth et al., 2018). Here, we examine the behaviour of liquid organic phase change paraffin wax as they state change in response environmental change. The interest in PCM façade is to choreograph the temporal behaviours of a set of architectural panels thereby controlling the interior environment behind them. The panels are differentiated in shape and volume in turn tuning the melting behaviours of the paraffin wax they contain. Thinner areas are more sensitive and therefore faster to melt, while thicker areas melt slower. Here, internal and external sensors are orchestrated to gather time stamped data interfaced with a predictive model that correlates the visual translucency as a function of shaping (Thomsen et al., 2020). This *post process* method allows different degrees of performance evaluation parametrising the design model directly. This is then interfaced with a set of second-process panels that correct and change initial design intent to be mounted after the first round of prototypical testing.

**Fig. 9:**
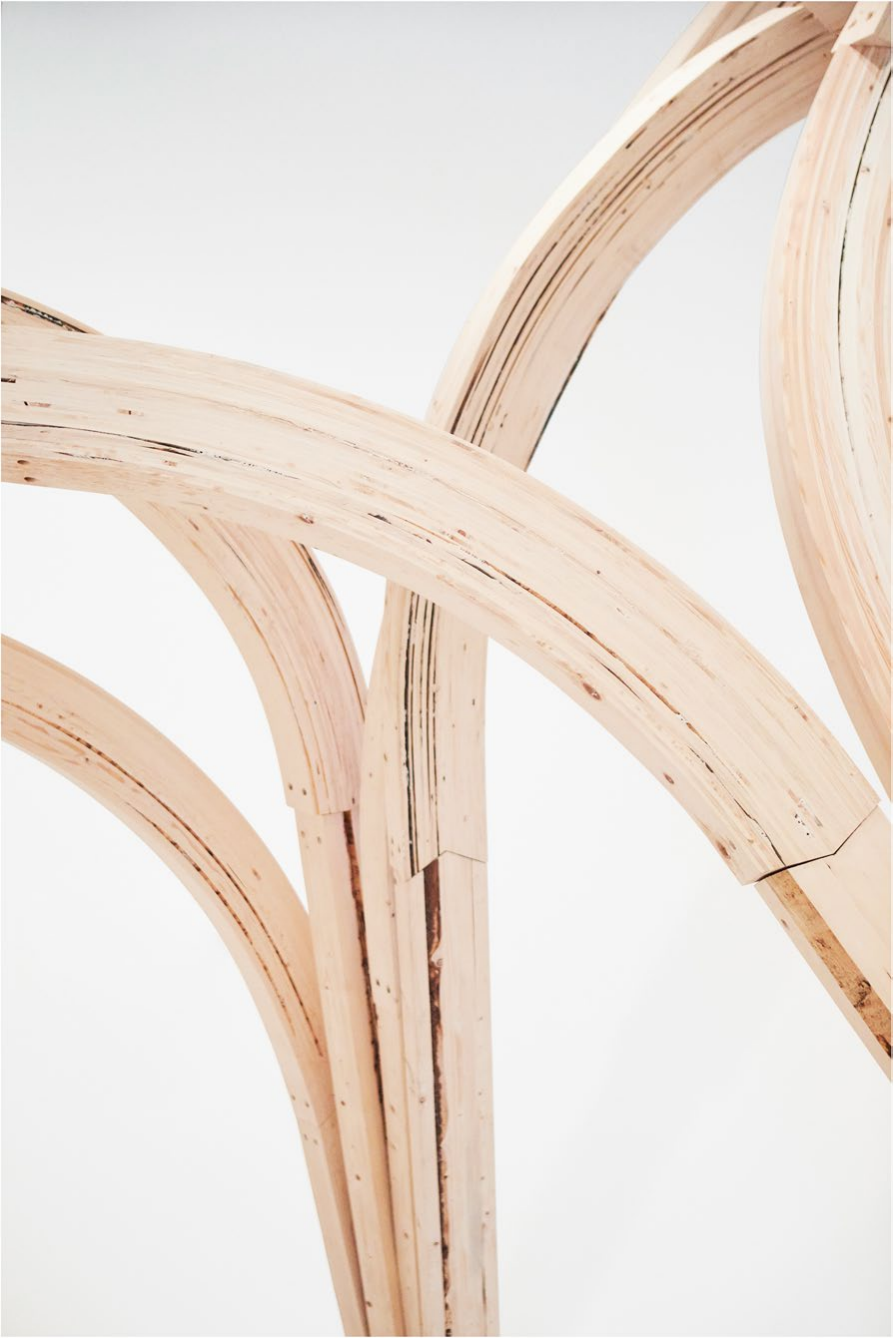
Rawlam, Bildmuseum, Umeå, Sweden. Detail of material heterogeneous as mapped on to design performance CITA, 2022

Integrating sensing is a fundamental building block in challenging the existing industrial paradigm of the standardised, the stable and the mass produced. To forward a paradigm of the highly contextualised site or use specific materials, functionally graded to specific localised performance, it is necessary to understand how the integration of sensing challenges existing fabrication set ups. Sensing allows us to understand the world as dynamic, as undergoing state changes instigated by environmental or performance changes. Moreover, it allows us to develop a representational framework that can capture, analyse and characterise these behaviours over time. As such, it challenges the foundational value proposition embedded in industrial architecture. Rather than corroborating design competition as the consolidated end point of design and construction, it opens new infrastructures to repetitive practices of amelioration, adaptation and maintenance (Fig. 9).

### Transferring the contributions of advanced computational modelling to a new bio-based material paradigm

The question addressed in this paper is how these methodological contributions can be understood as central steppingstones into an age of sustainability to engage with new renewable, regenerative and restorative material systems.

A central remit in transitioning into a sustainable design practice has been positioned around the innovation of new nature-based solutions. The European Green Deal and other Green Economy initiatives (A European green deal 2019; Chomsky & Pollin, 2020) builds upon a decade of research into renewable, regenerative and restorative design principles. Extending circular design with a bio-based agenda, they forward a model of material consumption based on reproduction and biodiversity advancing nature-based resource extraction processes. It is part of a larger framework of ecological thinking that has initialised the conceptualisation of our planet not as a place of infinite resources to be exploited, but instead as a network of interdependent systems construed in a finely tuned balance (Rockström et al., 2009; Thomsen et al., 2020).

Bio-based materials is one of the perspectives leading us from our current linear “take-make-dispose” models of production into renewable, regenerative and restorative methods. Beyond reconceiving the built environment as a bank for CO2 storage (King, 2017) directly impacting adverse greenhouse gasses, the materials themselves become part of larger cycles of use. These cycles can be understood as essentially metabolistic. As instances of biomass, bio-based materials are essentially cyclical as they pass through cycles of growth and decay at different intensities and temporalities (Thomsen & Tamke, 2022). As such, the bio-based material paradigm challenges the essentially waste based practices of industrialisation. Firstly, it suggests that materials are part of systems of return in the essentially closed loop planetary system. Secondly, it presents a new sense of agency as it positions us as part of the practices of growing and harvesting renewable materials. And thirdly, it points to the fundamentally transient temporality of architectural materiality.

However, such a material paradigm necessitates new methods of capturing the complexity of material systems as well as their systems of regeneration and inherent behaviour. Bio-based materials are fundamentally different to standardised industrial material systems. Shaped by growth cycles, bio-based materials are characterised by their high degree of structural heterogeneity. Incorporating mechanisms for water absorption and desorption, bio-based materials include hygroscopic behaviours changing in physical and mechanical properties as it responds to its environment. And, when considering bio-polymers and other bio-based composites they operate at distinctly different performances and life cycles than their fossil fuel based counterparts. Bio-based materials therefore ask us how to find new ways in which to design, analyse, specify and fabricate architecture. It is here that the central contributions of the computational design field and the emergence of the extended digital chain outlined above hold a particular role. In enabling a lower scale of material address, accommodating heterogeneity and instrumentalising behaviour, the last decade of research has created the methodological foundations for a transition into a bio-based material paradigm.

**Fig. 10:**
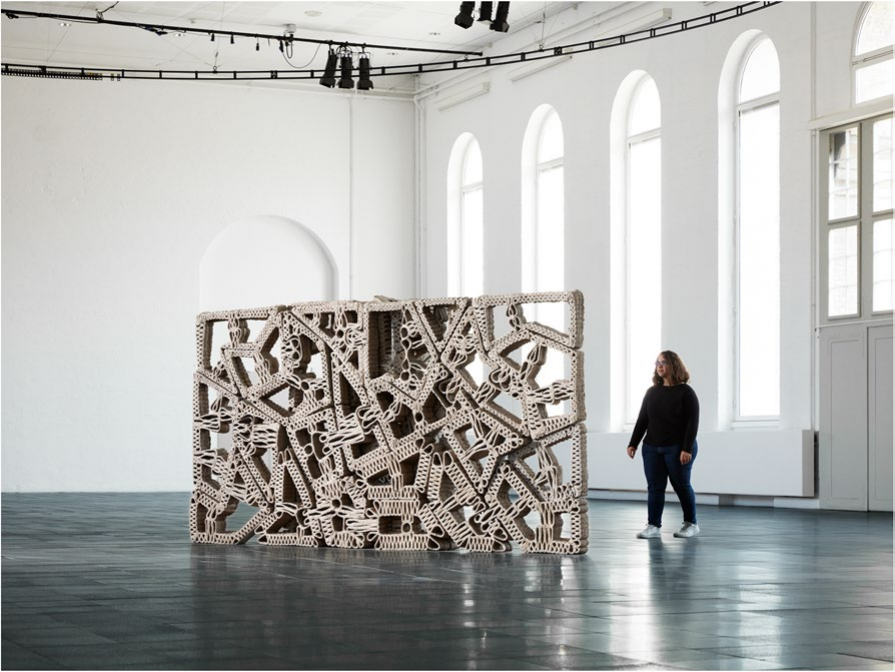
Predicting Response, 70% Exhibition, Royal Danish Academy. Predicting Response examines the 3D extrusion of cellulose reinforced biopolymer for architectural components. CITA 2022

The transferral of this methodological framework can be characterised across three central dimensions (Fig. 10).

#### Characterising the heterogeneous: challenging the axiom of standardisation and classification

The extended digital chain allows us to characterise localised performance of inherently heterogeneous materials. By integrating external sensing into the fabrication system that allows adaptive control allows the integration of a new sensitivity that can incorporate variation and mitigate failure in standardised systems. Industrialisation has dealt with heterogeneity through classification. A good example of this is the timber industry in which timber resources are classified along generalised qualities mapped to standardised performance criteria. This brackets the inherent heterogeneity of timber resource, its changing density, fibre directions, nots and wane and its resulting changes in performance within recognised criteria that can be generalised and shared. But it also results in an embedded wastefulness that our age can no longer afford to disregard. To truly enter a bio-based material practice we must find ways of interfacing the specificity of material resources with design and the specification of structural performance.

In CITA, this research direction is exemplified through the design integration of functionally graded glulam beams. Where the grading of engineered timber is already industry standard, then these strategies rely on the classification of timber at the sawmill. In Rawlam (Tamke, Svilans, & Thomsen, 2021; Tamke, Gatz, & Svilans, 2021) we examine how external sensing of material heterogeneity in the form of Computed Tomography (CT) scans can be interfaced with design intent in order to drive highly detailed processes of specification. Through a collaboration with timber CT scan developer MicroTec and Luleå University of Technology, CT scan data is used to drive highly specified understanding of resource and mapping local variation to performance at sub-part scale. This allows us to propose models for extending the value chain by widening the available resource for timber construction as lower quality timber can be upgraded from pulp-based products to products with greater degrees of longevity and therefore greater ability to store CO2. These prototypical methodologies allow us to speculate on future timber construction processes in which the heterogeneity of timber resource is understood as a quality advancing structural performance and guiding resource towards more intense use modes.

#### Capturing dynamic response, designing in time

**Fig. 11:**
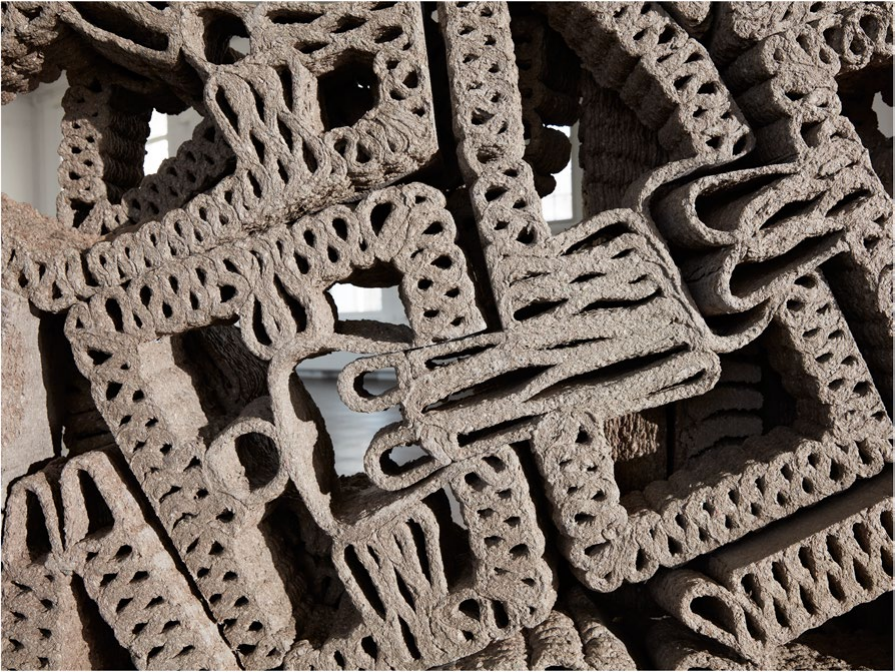
Predicting Response, Copenhagen. Detail of connection system. CITA 2022

The second dimension addresses behaviour. If the field of computational design has in its outset expanded structural thinking by instrumentalizing bending behaviour in standardised materials, then emerging methodologies from the field are understanding how these behaviours can be localised and steered as a result of design and specification. These methods gain new relevance in a bio-based material as the methods of characterising the active behaviour of material can be set in relationship to the specific behaviours of a given material system and allow its tuning. This methodological advancement is well exemplified in the field of bio-polymers in which the ‘recipe’; the way that binders, fibres and fillers are composed, can be freely composed in respect to given environmental concerns (the availability of resource, its locality or abundance) and performance criteria. The fundamental perception of materials as inherently designable follows a twentieth century percept in which radical innovation in polymer science drove a new class of materials. By situating this percept with a bio-based material paradigm, computational design is expanding these methodologies through the exploration of how inherent dynamic behaviours can be characterised and steered (Dahy & Knippers, 2017; Sanandiya et al., 2018), and how functional grading at element as well as material level can further tune the instrumentalization of these behaviours (Royo et al., 2015) (Fig. 11).

**Fig. 12:**
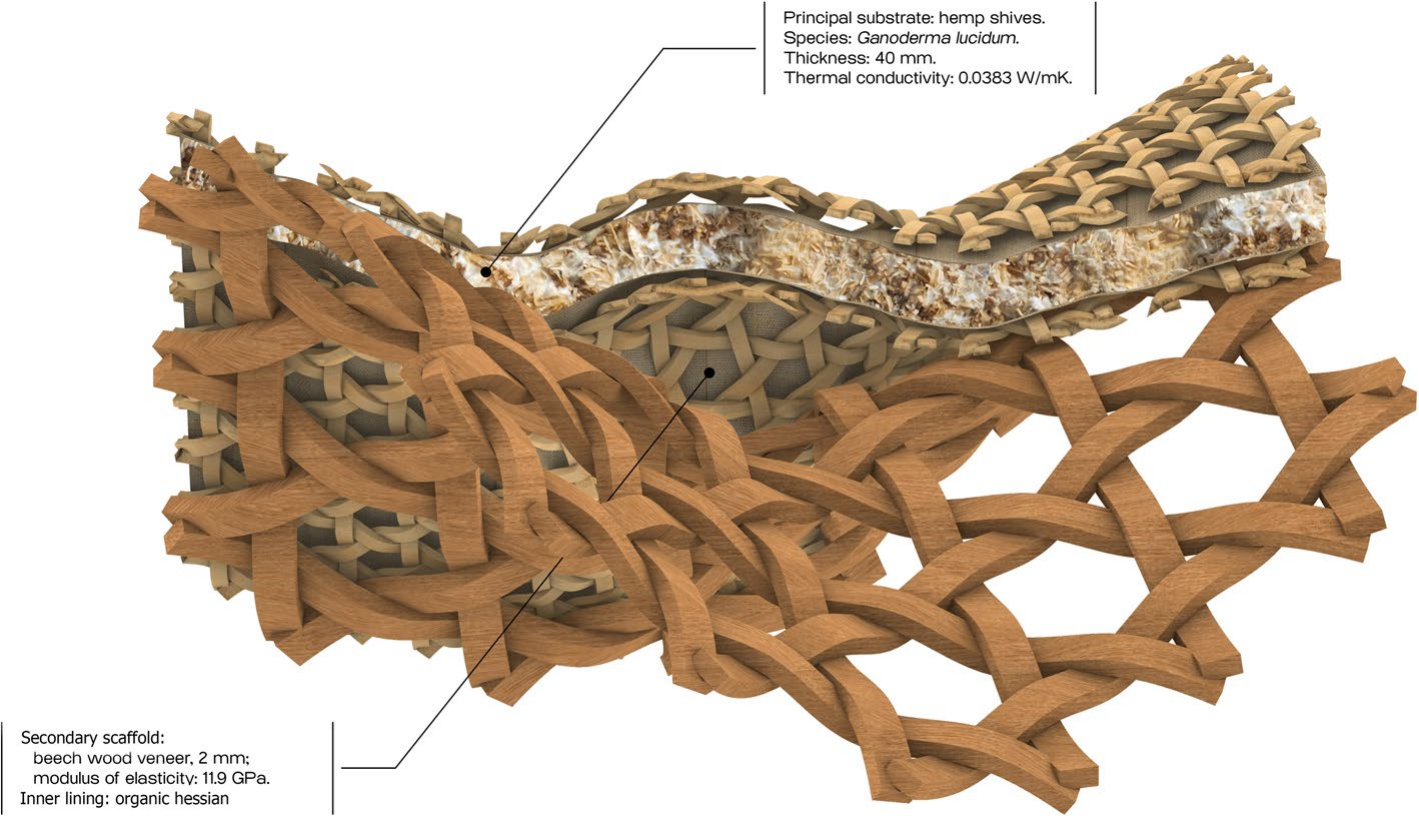
Detail of the FUNGAR construction concept with material composition and functional performance specifications derived from design objectives. CITA 2022

In CITA, we have examined this research direction through a series of projects into bio-polymer extrusion (Rossi et al., 2022). These projects develop methods for employing the orchestration of multiple sensor inputs drive multi-parameter time-stamped data in order to build a model that can characterise and predict drying behaviour of the model and interface these with design specification. At a fabrication level, our effort has been to build a monitoring framework that can drive meaningful sense data and correlate this to a statistical model to inform the design model and feedback to fabrication by correcting print path lines in respect to predicted outcomes. This production focus is paralleled by an environmental life-span focus. Here, the focus lies on how environmental impact onto the materials affects deterioration and decay. By combining external weathering tests with sped up weather chamber experiments, our aim is to query how material design can incorporate a temporal dimension and how the design of recipe, performance and geometry can specify a materials persistence. In this way we position bio-polymers as a means of thinking functional grading in respect to material life cycles, and integrating ideas of end-of-life scenarios into the designed building element. As such, we position grading as a seed to think differently about the durability, temporality and inhabitation of our built environment, moving from the fundamental value proposition of the building as persistent and enduring to the temporalized and deliberately destabilised (Fig. 12).

#### Designing for living materials

The third dimension opens new perspectives for designing with living materials. New directions in the digital design field are asking how methods of design steering can support the making of building elements from living materials. The initial interest in mycelium as a means of growing more or less specific building blocks that can be assembled more or less precisely (Heisel et al., 2017), is maturing into new questions of how the steering of growth environment (Ozkan et al., 2022) and strategies of substrate composition (Rigobello & Ayres, 2022; Rigobello et al., 2022) can lead to a steering of performance in new materials. While taking point of departure in craft practices of solid state fermentation, a key question revolves around methods of interfacing this practice with the existing extended digital chain. These advances define new ways of enhancing these methods by positioning fundamentally Anthropocene practices of growing and harvesting within the design chain. It places environmental control, nurture and crop as new concepts inside the design framework.

**Fig. 13:**
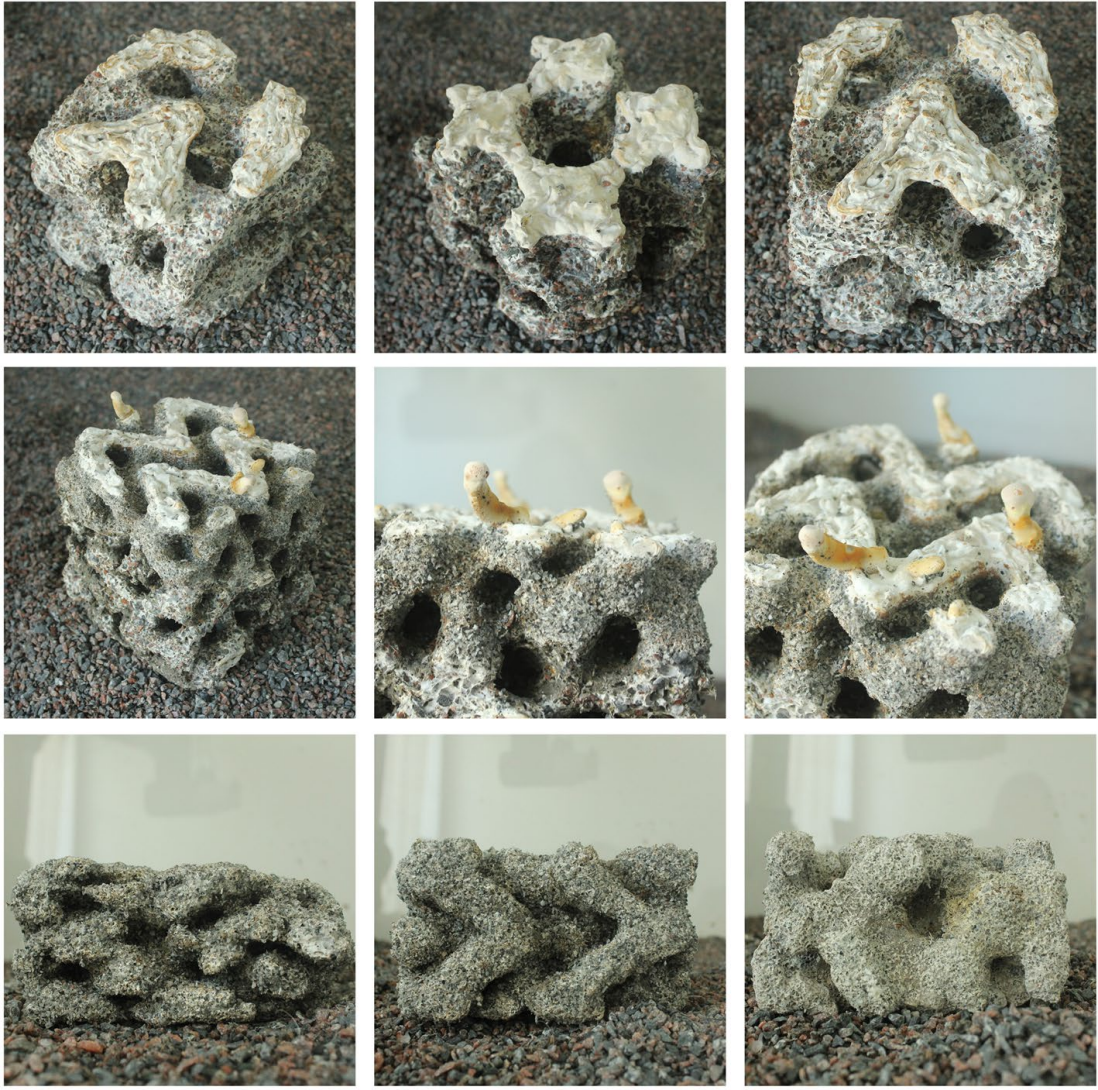
In Multi Materials Fabrication for Biodegradable Structures a two-phase multi-material fabrication process to create mycelium composite structures of higher porosity and complexity with speculated improvements in strength. First, cellulose pulp inoculated with mycelium is extruded. Then, each layer is filled by a secondary supporting material. This material, in the form of a gravel- and sand-slurry, acts as an inhospitable medium steering mycelial growth, additionally improving aeration to produce stronger structures. After an intermediate growth period, the secondary material, reusable in a closed-loop production model, is removed to reveal the fully-grown mycelium structure

In CITA, larger framework projects like FUNGAR (Adamatzky et al., 2019), experimental probes such as Multi Materials Fabrication for Biodegradable Structures and speculative design research projects such as *Restless Labyrinth* (Colmo & Ayres, 2020) are investigating how living materials can be interfaced with advanced modelling and fabrication, allowing for new ways of performance steering in grown materials, new material configurations, new structural assemblies and novel architectural proposition. In FUNGAR, research into substrate composition strategies are driving the instrumentalisation of digtally integrated performance specification within models of novel architectural assemblies (Fig. 13).

In Multi Materials Fabrication for Biodegradable Structures (Lim & Thomsen, 2021), the control of substrates is further probed with a geometric dimension in which two-phase fabrication processes steer internal cavities in turn tuning material performance. Here, the combining of robotically steered 3D extrusion with the layering of a secondary support material, allows us to compose and steer complex geometries with living systems.

Designing with for living systems challenges the way we understand fabrication in architecture. If computational design has opened up the remit of the standardised and idea of series to a new degree of sensitivity towards the localised and performance based, then including processes of growth and harvest into the design chain challenges us to build new understandings of the dynamics of living systems, their interactions with their containing environment and the transformations they undergo across their metabolic cycles, life spans and growth rates. These new questions rupture and expand our thinking of the heterogeneous and behaviour moving them from questions of distribution (in respectively space and time) to questions of evolution and interaction.

## Conclusion

The ambition of this paper is twofold. On the one hand the aim it to present a critique of the current direction of the field of computational design, on the other hand the endeavour to redirect the field’s central methodological contributions towards new questions emerging from an acknowledgement of our essentially Anthropocentric position. The critique questions whether the framework supporting industrialised building practice truly can innovate through a techno-centric model of optimisation of existing practice. By positioning the limitations of this practice, the paper has sought to challenge the central theoretical proposition of the field instead suggesting a transposition of the key contributions on to a bio-based material paradigm. This is not to see bio-based materials in isolation. They are part of a larger framework for sustainable material practice in which the recycled and the readapted take part. However, the bio-based material paradigm challenges us to reconsider our methodologies and see them in context of a new material identity characterised by the heterogeneous, the behaving and the grown and therefore expand the way we can think, design and make architecture.

A central position in the paper has been to present a series of disruptions that bio-based materials incur on industrialised building practice bringing forth the vision of an extended digital chain in which presumed linearity from design to production is dismantled in favour of cyclical methods defined by feedback between concurrent processes of specification and fabrication and nonlinear temporalised modelling practices driven by situated sense data. A core consideration is here how the embedded circularity of bio-based materials, their participation in the closed loop cycles growth and decay endemic to biomass, positions a bio-based architecture as something that is defined by its temporality and incorporates ideas of maintenance and end-of-life. This is de facto a theoretical change. Architecture has always incorporated maintenance. From the continual construction methods of artisanal and indigenous structures, to the highly formalised maintenance schedule of industrialised architecture, architecture is always part of cycles of repair, preserval and adaptation. But it is in contrast to the idealisation of architecture. In traditional architectural design culture as well as in industrialised practice, architectural design agency is assumed to end at the point of building completion. A bio-based material paradigm asks how architectural intent can extend its practices into a broader consideration of *life-span* and rethink ideas of maintenance as a means of continual construction, an enduring building practice.

## Data Availability

Not applicable.
